# An Evaluation of *¡Haz Espacio Para Papi!*, a Culturally Tailored Nutrition and Physical Activity Pilot Program for Mexican-Heritage Fathers

**DOI:** 10.3390/nu16081153

**Published:** 2024-04-13

**Authors:** Annika Vahk, Pablo Monsivais, Cassandra M. Johnson, Joseph R. Sharkey

**Affiliations:** 1 Nutrition and Exercise Physiology Program, Elson S. Floyd College of Medicine, Washington State University, 412 E. Spokane Falls Blvd., Spokane, WA 99202, USA; p.monsivais@wsu.edu; 2 Nutrition and Foods Program, School of Family and Consumer Sciences, Texas State University, 601 University Drive, San Marcos, TX 78666, USA; cassandra_johnson@txstate.edu; 3 School of Public Health, Texas A&M University, College Station, TX 77843, USA; jrsharkey@tamu.edu

**Keywords:** community nutrition, Latino fathers, rural, healthy behaviors, father–child program

## Abstract

Fathers are potential leaders of healthy behavior changes in their families. Culturally tailored programs are needed to support behavior changes within families, including Latino families; however, there have been few father-focused nutrition programs for Latino families. This study evaluated the immediate effects of *¡Haz Espacio Para Papi!* (Make Room for Daddy!; HEPP), a six-week, father-focused, family-centered program focused on nutrition and physical activity near the Texas–Mexico border. A modified stepped-wedge study design included a treatment group for the HEPP pilot and a wait-listed control group. Pre/post-tests included instant skin carotenoid scores, the self-reported dietary intake of fruits and vegetables (FV), and healthy dietary behavior scores (HDBSs). A 2 × 2 mixed analysis of variance evaluated changes in outcomes across time and between groups for 42 fathers with pre/post-test measures. There were no statistically significant changes in fathers’ VM scores and FV intake across time or between groups. Fathers’ HDBSs increased across time (*p* ≤ 0.01, 95% CI [0.23, 1.38]). Age, educational attainment, and the number of children living in the household did not have a significant effect on the program outcomes (*p* > 0.05). The HEPP program may guide the design of future father-focused nutrition interventions aimed at dietary behavior changes.

## 1. Introduction

Researchers have identified associations between paternal parenting practices, diet, and role-modeling and their children’s diet quality and dietary behaviors [1,2,3,4,5,6,7]. However, parental influences on healthy food practices and dietary behaviors have largely included mother–child programs as mothers spend more time at home and often bear greater responsibility for child care and food preparation compared to fathers [2,3,7]. With continual changes in modern family dynamics, fathers are spending more time interacting with their children in the home environment [2,7,8]. Fathers play an influential role on the dietary behaviors of their family members and can no longer be ignored in nutrition interventions designed for families. Currently, there are few published studies on family-centered nutrition interventions tailored for fathers [4,5,9,10,11,12], which calls for special attention to target and recruit male caregivers into programs [1,2,7,8]. In addition, little research targets underserved Latino fathers [9,10,11], who have expressed the desire to improve food practices and dietary behaviors within their homes and understand the importance of doing so for health purposes [8,13,14,15,16,17].

Family-based nutrition interventions targeting Latino children and families should include fathers for two important reasons. First, Latino fathers are role models and can serve as agents of behavior change within their households thereby positively impacting the whole family unit [1,2,8,10,12]. Interventions aimed at including Latino fathers benefit by designing culturally tailored programs, with highly regarded Latino cultural values which include *familismo* (familism), *respeto* (respect), and *colectivismo* (collectivism) [15,16,17]. These values convey the importance of the family working together as an entire unit instead of individualism. Family-based or centered interventions that integrate cultural values may improve acceptability by Latino fathers and effectiveness. Second, prior research indicates pronounced gender disparities in overall dietary quality and the dietary intake of fruits and vegetables (FV), with men typically showing substantially less-healthy dietary patterns than women [18,19,20]. For example, the average 2015 Healthy Eating Index (HEI) scores in Latino males aged 20–50 years who were residing with children in the household were significantly lower than Latina females (48.1 and 51.0, respectively) [18]. Latino males also reported a significantly lower energy-adjusted intake of FV (1.98 and 2.85, respectively) compared to Latina females (FV, 2.35 and 3.13, respectively). Along with the disproportionate burden of chronic disease among Latino adults, [21,22,23] these findings emphasize the need to support positive changes in dietary behaviors. Dietary behaviors encompass “all phenomena related to food choice, eating behaviors, and dietary intake/nutrition” [24]. Healthy dietary behaviors such as the dietary intake of FV have been associated with lower risks of cardiovascular disease, diabetes mellitus, and obesity [25,26].

Presently, the *¡Haz Espacio para Papi!* (Make Room for Daddy!; HEPP) program is the only family-centered nutrition and physical activity program designed for Latino fathers and specifically Mexican-heritage fathers in a border community [12,27]. Given that Latino fathers have not been intentionally included in nutrition interventions, the HEPP program fills a critical gap. This program was implemented in *colonias* (neighborhoods) in the Lower Rio Grande Valley of Texas, where persistent poverty and food insecurity make it difficult for residents to achieve or maintain a healthy diet [12,28,29]. The HEPP program included six weekly, culturally tailored sessions which focused on improving fathers’ dietary intake of FV and healthy dietary behaviors. We hypothesized that Mexican-heritage fathers participating in the program will report greater changes in program outcomes compared to the control group. The purpose of this study is to assess the effects of the six-week HEPP program for Mexican-heritage fathers on the (1) instant skin carotenoid scores via the Veggie Meter^®^ (VM), (2) self-reported dietary intake of FV, and (3) healthy dietary behavior scores (HDBSs) and assess the effects of age, educational attainment, and the number of children living in the household on changes in the program outcomes.

## 2. Materials and Methods

### 2.1. Study Design

This manuscript reports on secondary data from the HEPP pilot program, which used a modified stepped-wedge cluster randomized study design [12,27,30,31]. The HEPP pilot program was part of a larger community-engaged research project (*Salud Para Usted y Su Familia* [Health for You and Your Family]) in collaboration with *promotoras* (trained, native Spanish-speaking community health workers living and working in the community) [12,27]. *Promotoras* served as program interventionists and data collectors and are essential to effective interventions for Latino populations [12,27,31]. Prior to the start of the program, *promotoras* completed approximately 537 h of in-person training activities which equipped them with knowledge and skills needed to instruct cooking activities and provide nutrition education. The training activities included games, discussions, demonstrations, role-playing, mini presentations, guided and self-study activities, observations with feedback, and booster training [30]. The program design was guided by the literature, theories, formative research, and pre-testing and designed simultaneously in English and Spanish [12].

**Figure 1:**
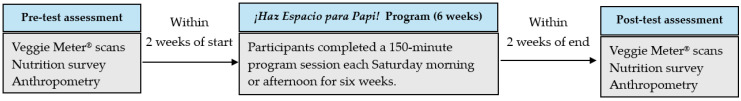
The timeline for Mexican-heritage fathers’ participation in the *¡Haz Espacio para Papi!* (HEPP) Program. This figure shows the outcomes assessed in this manuscript pre-test and post-test. While anthropometry was assessed, this was not a program outcome. Data collection occurred at baseline (pre-test), within two weeks of the start of the program and program conclusion (post-test) within two weeks of the end of the program.

In the stepped-wedge design, five clusters (groups of fathers) were randomly assigned to start the program (treatment group) or serve as the wait-listed controls [32]. Two weeks prior to group 1 starting the program, *promotoras* collected pre-test (baseline) data for groups 1 and 2 (indicated by orange blocks at week 0 in Table 1). In step 1, group 1 started the program and group 2 served as the controls (blue and gray blocks in Table 1). Within two weeks of program completion, post-test data were gathered from both groups 1 and 2 (indicated by orange blocks at week 10 in Table 1). This design permitted steps of delayed treatment or wait-listed program participants for groups 2–5. The benefits of this design included minimizing the resources needed for program implementation and maximizing the opportunities for all enrolled participants to receive the program. Figure 1 shows the timing of pre/post-test assessments and HEPP programs for each group. Groups 1–4 participated in the program between July 2019 and January 2020 in sequential order. Group 5 began the program in February 2020; however, due to the onset of COVID-19 in early 2020, the program was discontinued.

### 2.2. Participant Recruitment

Prior to recruitment, the research team identified 16 clusters or defined geographical regions within Hidalgo County in the Lower Rio Grande Valley of Texas [12,30,31]. The geographic clusters of *colonias* were similar in population, sociodemographic characteristics, environmental boundaries, and size. Families living in border *colonias* experience high rates of poverty, food insecurity, and diet-related chronic diseases and have poor eating behaviors (e.g., a low intake of FV) [28,29,30]. *Promotoras* recruited families using word of mouth, flyers, in-person methods, and re-contacting families who had previously participated in other observational studies one to two years prior to HEPP [12,30,31]. The inclusion criteria for the HEPP program included families with (1) Mexican-heritage parents (21 years or older), (2) a parent living with a spouse or partner and a child (9–11 years) in the geographical area for at least 12 months, and (3) the ability to fully participate in a six-week program and complete all home-based assessments. Families were excluded if a parent or child had any physical limitations or food allergies.

Prior to participant recruitment, the HEPP pilot program and materials were reviewed and approved by the Texas A&M University Institutional Review Board (protocol numbers 2014-0825D and 2019-0750). All fathers provided informed consent. *Promotoras* facilitated the informed consent process in Spanish and English and included visual aids with graphics. Written materials were created at or below the fifth-grade reading level. As an incentive, participants were offered USD 100 cash if the entire family triad (father, mother, child) completed pre-test assessments. A USD 200 cash incentive was offered for group 1 participants for attending all six program sessions, who did not serve as wait-listed controls. Participants in groups 2–4 received a larger USD 250 cash incentive because they served as wait-listed controls. Also, families that attended all program sessions were gifted the full kitchen toolkit used throughout HEPP. Lastly, participants received USD 200 cash incentives for completing post-test assessments.

### 2.3. Program Delivery and Components

A team of *promotoras* implemented the program mainly in Spanish, though *promotoras* engaged with participants in English and distributed materials in English as appropriate [12]. The delivery was face-to-face and in a group format with six weekly group sessions at 150 min each [12]. The nutrition curriculum incorporated nutrition principles from the *Dietary Guidelines for Americans* and MyPlate with an emphasis on fruits and vegetables [26]. Within the general population, low fruit, vegetable, and fiber intakes have been reported [19,20,26]. Therefore, the program curriculum focused on increasing the intake of fiber-rich foods, specifically the consumption of FV. Additionally, previous research has established the importance of including cultural traditions for promoting the program adherence and participation of Latino families [12,13,15,16,17,33,34]. The HEPP program included traditional foods, food practices, games, and activities.

Each session began with tasting recipes for the families, which were prepared by the *promotoras* and included an abbreviated nutrition education lesson. Fathers participated in interactive activities (e.g., nutrition education lessons, cooking, and goal setting), which promoted positive family dynamics and skill-building related to healthy dietary behaviors (e.g., cooking with vegetables at home). Fathers served as both role models and mentors for their children during the activities. Each group session ended with the *promotoras* presenting at-home activities for families to complete alongside instructional guides and tools, educational materials, and self-monitoring worksheets to encourage co-participation in food-related activities (e.g., cooking healthy recipes with their child). This supported increases in families’ dietary intake of FV and facilitated families cooking healthier versions of traditional meals (e.g., chicken tostado) together.

### 2.4. Data Collection and Measures

*Promotoras* collected all interviewer-administered data in the home two weeks prior to the start of the program (pre-test) and within two weeks after the completion of the program (post-test) [12,31]. The assessments included anthropometry, VM scans (Longevity Link, LLC, Salt Lake City, UT, USA), and nutrition surveys. *Promotoras* measured weight using digital scales and height using a stadiometer. Body mass index (BMI) was calculated using weight (kg) divided by height (m^2^). VM scans were used as a biomarker for the dietary intake of FV. The VM uses reflection spectroscopy to detect and quantify the concentration of dermal carotenoids, which are found in colorful FV, such as carrots and red peppers [35,36]. The VM tool has been shown to provide a rapid, non-invasive, and objective method for estimating FV intake across diverse populations, including among low-income Hispanic and Latino adults and children [37,38,39,40,41,42]. Current research indicates that the reference period for the VM is about the past eight weeks [35,39,40]. In addition, *promotoras* collected survey data on covariates, confounders, and outcomes with two interviewer-administered surveys: (1) a sociodemographic survey and (2) nutrition survey (File S1: HEPP Father Nutrition Survey). The sociodemographic survey collected information on fathers’ age, educational attainment, and marital status and the number of adults and children living in the household and ages of household residents. The nutrition surveys included Likert scale questions regarding diet and dietary-related behaviors. *Promotoras* used response grids as a visual aid during nutrition survey data collection. The response grids were provided to ease challenges in reporting behaviors, such as the frequency of eating fresh fruit, 100% fruit juice, white potatoes, lettuce, and other vegetable categories. The frequencies were reported for each of the food categories and for the dietary-related behaviors (e.g., prepare snacks at home for your children using FV). The reference period for the dietary intake of FV and dietary-related behaviors was the week prior.

### 2.5. Treatment of Variables

The HEPP program aimed to increase fathers’ dietary intake of FV through co-participation in desirable dietary behaviors (e.g., cooking), which promote healthy dietary behaviors within the household (e.g., prepare snacks at home for your children using FV). Therefore, for statistical analysis purposes, a healthy dietary behavior score (HDBS) was created for each father by summing the responses of all nine dietary behavior questions using a 3-point Likert scale. The theoretical range for HDBSs was 0–18 based on the 0, 1, or 2 code assigned per response category (never, once-in-a-while, and almost every day, respectively).

### 2.6. Analytic Sample

**Figure 2:**
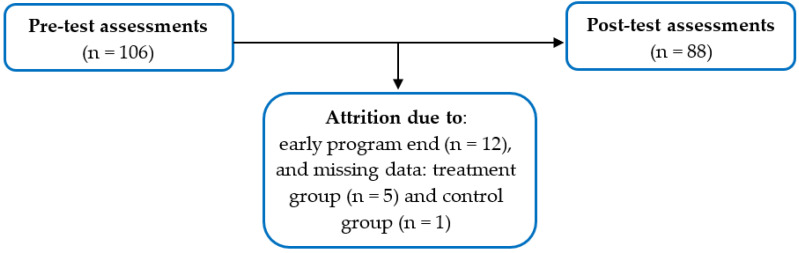
Flowchart demonstrating pre- and post-test assessments and attrition of HEPP participants. Both treatment and wait-listed control participants were included in totals.

Fifty-nine fathers consented to participate in the program and completed pre-test assessments and were divided into groups 1–5 [12,27,31]. Four groups of 10–12 families completed the program in four sequential steps between July 2019 and January 2020. Due to the onset of the COVID-19 pandemic, families in group 5 only attended two sessions before the program was terminated. Therefore, participants from group 5 (*n* = 12) were excluded from data analyses. Five additional fathers from groups 1–4 were excluded from data analyses due to missing post-test data, leaving a total of 42 fathers in the treatment group. Forty-seven fathers from groups 2–5 served as wait-listed control comparisons during the study period. However, one control comparison with missing post-test data was removed from data analyses. Overall, a total of 106 pre-test and 88 post-test assessments were gathered from the treatment group and control comparisons (Figure 2). The retention of the treatment group participants and control comparisons at the post-test assessment was 83%.

### 2.7. Statistical Analyses

All statistical analyses were conducted using Stata software (StataCorp. Stata Statistical Software: Release 18. College Station, TX, USA: StataCorp LLC), and the alpha level was set at 0.05 [43]. Descriptive statistics were computed for all data and are presented as the mean ± standard deviations. Frequencies and percentages were presented for sociodemographic information. Skewness and kurtosis were assessed for all data to ensure the data were within acceptable skewness and kurtosis ranges. Schmider et al. found no significant differences between tested distributions of data, suggesting the analysis of variance (ANOVA) test is robust to type I and type II errors when data distribution falls within tested skewness (−2 and +2) and kurtosis (−9 and +9) ranges [44]. Levene’s test of sphericity was used to assess the homogeneity of variance [45]. The *F* statistic, degrees of freedom (*df*), *p* values, and effect sizes (*η*^2^) or 95% confidence intervals (CIs) were reported for each statistical analysis.

This pilot study did not have a *per protocol* analysis set *a priori;* therefore, an intention-to-treat analysis approach was used which included all 42 fathers with pre- and post-test measurements regardless of low program dose or dropout [45]. A 2 × 2 ANOVA test was used to compare fathers’ weekly average VM scores, self-reported dietary intake of FV, and HDBSs across time and between groups [45]. A *post hoc* analysis using a Šidák adjustment for multiple pairwise comparisons was conducted when findings were significant. Paired samples *t*-tests were also included to investigate within-group mean changes in program outcomes during the intervention period (difference = post-test − pre-test).

A sensitivity analysis was conducted due to suspected post-test VM scan measurement errors in two fathers from the treatment group and one father’s pre-test score from the control group. This analysis allowed for the comparison of VM results across the analyses with and without the three data points [45]. Furthermore, to investigate the potential efficacy of the program, an additional 2 × 2 ANOVA analysis excluding fathers who attended less than 50% of the program sessions was conducted (*n* = 7; an analytic sample of *n* = 35).

Additional analyses were conducted to determine if potential moderating variables had main or interacting associations with the intervention effect. Moderator variables change the size or direction of the relationship between the intervention and program outcomes [46]. Three variables, age, educational attainment, and the number of children living in the household, were stratified into two subgroups based on the median value and used in analyses [47,48]. Lastly, a parametric Pearson correlation coefficient was calculated to explore the relationship between fathers’ weekly average VM scores and self-reported dietary intake of FV [45].

Since the HEPP program was a pilot study [12], an *a priori* power analysis was not calculated for the sample size. Due to the small effect sizes (*η*^2^) reported in the findings, a *post hoc* power calculation was completed to determine the likelihood of a type II error [45]. Sample size is one of the determinants of the statistical power of a test, and the G*Power (v.3.1.9.7) calculator was used for a *post hoc* power analysis [49]. The findings showed that for a 2 × 2 mixed ANOVA, the minimum sample size required to reach a statistical power of 80% was 34 participants. For our sample of 42 participants measured across two different time points, the statistical power was 88% to detect a significant program effect. There was a small probability of committing a type II error with a medium effect size (*F* = 0.25).

## 3. Results

### 3.1. Sociodemographic Characteristics of HEPP Program Participants

All fathers participating in the HEPP program were of Mexican heritage. Fathers in the treatment group were also represented in the wait-listed control comparison group. Therefore, the treatment and control comparison groups were similar in pre-test (baseline) sociodemographic characteristics (Table 2). Fathers in the treatment group had an average age of 39.0 ± 7.3 years, with a mean BMI of 30.5 ± 5.2 kg/m^2^ (Table 2). The number of household members ranged from 2 to 5 adults and 2 to 13 children with average ages of 36.6 ± 11.4 years and 9.4 ± 4.2 years, respectively.

Of the 42 fathers in the treatment group, 31 fathers (73.8%) attended all six program sessions, and 4 fathers (9.5%) attended five sessions. Two fathers (4.8%) attended two sessions, and two fathers (4.8%) attended only one session. Lastly, three fathers (7.1%) did not attend any sessions, however they completed pre- and post-test measurements.

### 3.2. Dietary Trends across Time for Treatment and Wait-Listed Control Comparison Groups

The treatment group’s weekly average VM scores were similar between pre- (288.6 ± 45.7) and post-test (289.8 ± 60.6). The treatment group’s total weekly average FV intake increased from pre- to post-test (9.2 ± 4.9 and 9.9 ± 4.1, respectively; Table 3). A similar trend was observed for the treatment group’s average HDBSs from pre- (6.5 ± 2.1) to post-test (6.9 ± 1.6). The control group’s weekly average VM scores, self-reported dietary intake of FV, and HDBSs also increased from pre- to post-test. Paired samples *t*-tests displayed non-significant within-group mean changes for each program outcome during the intervention period for both groups (*p* > 0.05; Table 3).

### 3.3. Comparisons of Fathers’ Objectively Measured Dietary Intake of Fruits and Vegetables Using Veggie Meter^®^ Scores across Time and Between Groups

A simple main effects analysis showed group and time did not have a significant effect on VM scores (*p* = 0.27, *p* = 0.72, respectively; Table 4). There was no statistically significant interaction between the effects of time and group on VM scores (*F*_(1,86)_ = 0.06, *p* = 0.81, *η*^2^ < 0.01; Table 4). A sensitivity analysis, which excluded three erroneous VM measures (two scores from the treatment and one from control comparison group), also revealed no statistically significant interaction between the effects of time or group on VM scores (*F*_(1,83)_ = 0.26, *p* = 0.61, *η*^2^ < 0.01).

### 3.4. Comparisons of Fathers’ Weekly Average Self-Reported Dietary Intake of Fruits and Vegetables across Time and Between Groups

A simple main effects analysis showed group and time did not have a significant effect on FV (*p* = 0.36, *p* = 0.08, respectively; Table 4). There was no statistically significant interaction between the effects of time or group on fathers’ weekly self-reported dietary intake of FV (*F*_(1,85)_ = 0.58, *p* = 0.45, *η*^2^ < 0.01; Table 4).

### 3.5. Comparisons of Fathers’ Weekly Average Healthy Dietary Behavior Scores across Time and Between Groups

A simple main effects analysis showed group did not have a significant effect on HDBSs (*p* = 0.25; Table 4). A simple main effects analysis showed time had a significant effect on HDBSs (*F*_(1,86)_ = 8.88, *p* = <0.01, 95% CI [0.22, 1.05]). Post hoc analyses using a Šidák adjustment for multiple pairwise comparisons revealed fathers’ HDBSs in the control group significantly increased from pre- to post-test (*p* ≤ 0.01). There was no statistically significant interaction between the effects of time or group on fathers’ HDBSs (*F*_(1,86)_ = 0.70, *p* = 0.41, *η*^2^ < 0.01; Table 4).

### 3.6. Sociodemographic Effects on Program Outcomes

Three categorical variables were included in the analysis to explore their potential moderating influence on program effects. There were no significant three-way interaction associations of age, educational attainment, and the number of children living in the household between fathers’ weekly average VM scores, time, and group (Table 5). This was similar for the self-reported weekly average dietary intake of FV, time, and group. There was a significant main association of time with age, educational attainment, and the number of children living in the household for fathers’ weekly average HDBSs (*p* ≤ 0.01, *p* ≤ 0.02, and *p* = 0.01, respectively). There was no interaction between group and age for HDBSs (*p* = 0.37). However, healthy dietary behavior scores significantly increased from pre- to post-test in control comparisons who were younger (*estimate* = 1.05, 95% CI [0.18, 1.92], *p* = 0.02). There was no significant interaction between educational attainment and group (*p* = 0.20), but HDBSs significantly increased between pre- and post-test for control comparisons with higher educational attainment (*estimate =* 1.73, 95% CI [0.56, 2.90], *p* ≤ 0.01). There was no significant interaction between the number of children living in the household and group (*p* = 0.17). Of note, HDBSs significantly increased in control comparisons with fewer children living in the household (*estimate =* 1.13, 95% CI [0.32, 1.93], *p* ≤ 0.01).

### 3.7. Analyses with Attrition

Sociodemographic information for the treatment and control groups for the analysis with attrition (*n* = 35) is presented in Table S1. Program outcomes had non-significant upward trends in both groups and paired samples *t*-tests showed a non-significant within-group mean change for each program outcome during the intervention (*p* > 0.05; Table S2). The analysis with attrition also presented non-significant ANOVA findings for the effects of group, time, and interaction between the effects of time or group on fathers’ VM scores and FV intake (*p* > 0.05; Table S3). A simple main effects analysis showed group did not have a significant effect on HDBSs (*F*_(1,86)_ = 0.67, *p* = 0.41, *η*^2^ < 0.01; Table S3). A simple main effect analysis showed time had a significant effect on fathers’ HDBSs, with the control group’s HDBSs significantly increasing across time (*F*_(1,79)_ = 6.36, *p* = 0.01, *η*^2^ < 0.02). There was no statistically significant interaction between the effects of time or group on fathers’ HDBSs (*F*_(1,79)_ = 1.03, *p* = 0.31, *η*^2^ < 0.01; Table S3). The addition of potential moderators (age, educational attainment, and the number of children living in the household) to the analysis presented similar subgroup effect findings as those reported for the intention-to-treat analysis (Table S4).

## 4. Discussion

The main findings were that the *¡Haz Espacio para Papi!* (Make Room for Daddy!; HEPP) pilot program showed a significant increase in healthy dietary behavior scores (HDBSs) for wait-list controls but not for the treatment participants. The HDBS, used in this current study, largely captures healthy dietary behaviors that include both father and child (e.g., *in the past week, how often did you prepare snacks at home for your children with fruits and vegetables?*). Throughout the intervention, fathers and children co-participated in interactive cooking lessons and program activities which are important for establishing and transferring healthy dietary behaviors into the household. The reasons for non-significant findings in treatment participants may be related to the measurement of program outcomes or the relatively short program duration, which may have not been long enough to establish new dietary behaviors. For example, some fathers may have experienced long or changed employment hours which limited free time to prepare home-cooked meals with children. Also, Latino gender roles between households may have impacted fathers’ behaviors. Traditionally, Latina mothers are expected to take care of house responsibilities including cooking and preparing meals for the family, and as a result, fathers may have participated less in the specific healthy dietary behaviors captured by the study’s nutrition survey (e.g., cooking and preparing meals/snacks) [8,12,16,50]. Despite being instructed to maintain normal daily habits throughout the study, it is common for control participants to make changes in daily dietary habits [51]. Controls’ reactive behaviors through simple exposure to the study and data collection meetings may have introduced experimental bias in this study and may be an explanation to the significant changes in HDBSs across time and the lack of significant findings between groups.

Socioeconomic status, including educational attainment, is associated with dietary behaviors among adults [47,48] and important for the understanding of its influence on dietary behaviors among Latino populations [31,48]. This study examined potential moderators of age, educational attainment, and the number of children living in the household. Interestingly, for control comparisons, younger fathers, those with higher educational attainment, or with fewer children had significantly greater HDBSs post-test. Explanations for these findings are difficult to provide as it was anticipated for controls to experience no significant changes in program outcomes throughout the study. Again, the lack of blinding and exposure to study assessments may have prompted reactive behaviors by controls [51].

A few points warrant discussion. First, this study found no significant differences between groups or across time for the objectively measured dietary intake of FV using VM scans or subjectively measured FV intake using surveys. Other family-based programs for Latino families have found significant improvements in the dietary intake of FV, but the programs were designed and implemented in different contexts, for different samples of fathers, and with different doses and program lengths [10,52,53,54]. Of note, our analysis with attrition, which excluded fathers (*n* = 3) who received a lower program dose (less than 50%), did not produce different findings from the main analysis. The six-week HEPP program may have been too short to establish new dietary behaviors or capture changes in skin carotenoids (via VM scans), which take approximately eight weeks to deposit [35,39,40].

Second, fathers’ weekly average VM scores pre- (288.6 ± 45.1) and post-test (289.2 ± 60.1) were higher compared to previous research with predominately Hispanic and African American adults [37,38], and there were positive trends in the self-reported FV intake post-test. However, Latino fathers’ self-reported FV intake was lower than the recommended levels [26]. The findings from this study can be used to strengthen existing nutrition assistance programs and consider new policies or programs that increase fruit and vegetable access for Latino families.

Third, the retention at post-test assessment was relatively high (83%) compared to similar programs for Latino families with retention between 46% and 75% [9,10,11,52,54,55]. The design and implementation of the HEPP pilot program involved extensive engagement with *promotoras* [12,30,31], which promoted retention [33]. An advantage of utilizing a *promotora*-based approach is the rapport and trust that is built between the interventionists and participants, [30,33] which is imperative for the recruitment and retention of Latino families from low socioeconomic communities, where trust and safety is extremely important [12,16,33,56]. In this pilot study, the relatively high retention and participant satisfaction based on qualitative data (not reported in this study), indicated the HEPP program was feasible, positively accepted, and valued for Mexican-heritage fathers.

In addition, culturally tailoring the program for Latino families, and recognizing fathers as coparents and partners, supported retention [12]. Previous healthy lifestyle programs tailored for Latino participants showed high participation and satisfaction with program offerings [11,52]. Specifically, incorporating cultural values and beliefs such as familism, collectivism, and respect is essential for Latino fathers to consider a program as benefiting their entire family unit while also reinforcing their cultural values [15,16,17,53,55]. The HEPP pilot program focused on father–child co-participation in nutrition activities, which reinforced family dynamics and supported Latino values [12]. The program also incorporated healthier versions of traditional recipes and foods which were important to families and were inclusive of the food preferences of each family member (e.g., meat-filled tacos for fathers, vegetable pinwheels for children) [12]. Families were provided with monetary and gift incentives, which may have motivated families to continue attending HEPP sessions. However, it is important to understand that incentives alone may not promote the retention of Latino families in programs [9,33,34]. Reported barriers to family-based program participation by Latinos include the lack of childcare for non-participating children and transportation to program site [16,33]. The HEPP program offered both, which may have also bolstered retention. Due to the fathers’ work demands, offering program sessions on weekends also encouraged recruitment and program engagement [9,12,27]. In addition, the use of a stepped-wedge study design was advantageous for enabling all participants to receive the program benefits while also reducing the number of resources needed to provide the program [27,32].

Due to the pilot nature of the HEPP program, there are several limitations to take into consideration. Although the *post hoc* power analysis determined there were enough fathers to detect statistically significant effects, dietary behavior changes take time. A longer program may have yielded alternative findings, and future research might consider extending the program length beyond six weeks. Despite the pilot testing of the nutrition survey for quality and effectiveness in a small group of Mexican-heritage families, the survey used in this study had not been previously validated in the Latino population. Furthermore, the self-reported dietary intakes and healthy dietary behaviors are subject to social desirability and recall bias, which may have affected data accuracy. To this point, in this study, there were no significant correlations between VM scores and the self-reported dietary intake of FV pre- (*r* = −0.24, *p* = 0.12) and post-test (*r* = −0.14, *p* = 0.39). This finding indicated a discord between the objective and subjective tools used to estimate fathers’ weekly dietary intake of FV. Previous studies have found positive relationships between skin carotenoid measurements and more rigorous, validated dietary surveys with 24 h and 30-day reference periods [37,38].

There are several reasons for a lack of correlation between the objective and subjective estimates of FV intake. These include different reference or exposure periods between the VM tool and nutrition survey (past eight weeks and week, respectively), and the accuracy and reliability of the VM scans may have been affected by using the VM in a community and not a clinical or lab setting. In addition, this program was an early adopter of the VM tool and was completed before the methodological recommendations for the VM were published [36]. Environmental factors, such as temperature or lighting may have influenced the accuracy and reliability of VM scores [36]. Future recommendations include completing the VM assessments in a controlled environment (i.e., laboratory or climate-controlled indoor space) instead of outdoor settings [36]. Three fathers had erroneous post-test VM data which may have affected the study findings. Consistent data collection procedures (e.g., tool calibration, finger preparation, etc.) are necessary to ensure the validity and reliability of measurements [36]. Another reason may be related to the amount of time required for the deposition of carotenoids in the skin (e.g., the reference period of the past eight weeks), which may have masked or under-represented fathers’ dietary intake of FV across the six-week program [35,39,40]. Seasonality may have also been a factor and influenced access to FV for fathers across groups [57]. Lastly, this study specifically targeted low-income Mexican-heritage families living in Texas–Mexico border communities; therefore, the results may not be generalized to Latino fathers of different heritages living in other locations where communities, resources, and environments may differ.

## 5. Conclusions

Despite the lack of significant findings for the dietary intake of fruits and vegetables and healthy dietary behaviors, this study demonstrated the feasibility of implementing a father-focused, family-centered program for Mexican-heritage families living in a rural setting along the Texas–Mexico border and may be used to inform the development, implementation, or evaluation of future programs. Additional recommendations for future research include extending the program duration beyond six weeks to allow for adequate time for detecting changes in skin carotenoid levels or self-reported healthy dietary behaviors. Lastly, future research may benefit from measuring additional factors outside of a program setting such as the household environment and food accessibility and affordability as influencing factors of multi-level behavior change in Latino households to investigate potential effects on participants’ dietary intake and behaviors.

## Figures and Tables

**Table 1:** The modified stepped-wedge study design demonstrating the timeline of pre- and post-test assessments, and intervention and control periods for five clusters (groups of fathers) enrolled in the *¡Haz Espacio Para Papi!* (HEPP) program.

	Weeks										
**Clusters**		**0**	**6**	**10**	**16**	**20**	**26**	**30**	**36**	**40**	**42**
	**1**										
	**2**										
	**3**										
	**4**										
	**5**										

Orange blocks indicate each groups’ assessment periods (pre/post-test). Blue blocks indicate the time each group participated in the six-week HEPP program. Gray blocks indicate the wait-listed control period of each group during the study. Group 5 completed only two weeks of the program, and no post-test assessments were completed.

**Table 2:** Baseline sociodemographic characteristics of 42 Mexican-heritage treatment participants and 46 wait-listed control comparisons participating in a six-week father-focused family-centered nutrition program.

Sociodemographic Characteristic	Treatment (*n* = 42)	Control Comparison (*n* = 46)
	Mean ± s.d. (Range) or *n* (%)	
Age	39.0 ± 7.3 (26–59)	38.7 ± 8.9 (26–58)
Body mass index ^a^	30.5 ± 5.2 (22.1–46.1)	31.3 ± 5.5 (22.1–49.7)
Marital status		
Married	27 (64.3)	25 (54.4)
Living with spouse—not married	15 (35.7)	21 (45.7)
Education attainment		
Did not go to school	1 (2.4)	1 (2.3)
Some school	29 (69.1)	34 (73.9)
GED or HS diploma	10 (23.8)	9 (19.6)
Some college	2 (4.8)	1 (2.2)
College degree	0 (0.0)	1 (2.2)
Total residents in household	7.3 ± 2.4 (4–16)	7.1 ± 2.0 (4–13)
Number of adults ^b^	2.6 ± 1.0 (2–5)	2.7 ± 1.0 (2–6)
Number of children ^c^	4.7 ± 2.2 (2–13)	4.4 ± 1.9 (2–9)
Ages of adults in household (years of age)	36.6 ± 11.4 (18–79)	37.3 ± 12.3 (26–77)
Ages of children in household (years of age)	9.4 ± 4.2 (3 mo–17)	9.3 ± 4.2 (3 mo–17)

Abbreviations: GED, general education development and HS, high school. *Promotoras* gathered pre-test (baseline) sociodemographic characteristics from program participants and wait-list control comparisons two weeks prior to participants starting the program. ^a^ Body mass index was calculated using kg/m^2^. ^b^ Adults were defined as ≥18 years of age. ^c^ Children were defined as ≤17 years of age.

**Table 3:** Descriptive statistics of dietary trends and within-group mean change from pre- to post-test.

Measure	Treatment (*n* = 42)				Control Comparison (*n* = 46)			
	Pre-Test	Post-Test	Mean Change	*p* Value	Pre-Test	Post-Test	Mean Change	*p* Value
**Total fruit ^a^**	3.9 ± 2.4 (0–10)	4.0 ± 2.3 (1–10)	0.12 ± 1.9	0.69	3.5 ± 3.2 (0–16)	3.7 ± 2.4 (0–10)	0.13 ± 3.6	0.81
Fresh fruit	2.7 ± 1.6 (0–7)	2.8 ± 1.5 (0–8)	0.17 ± 1.5	0.49	2.3 ± 2.6 (0–16)	2.5 ± 1.6 (0–7)	0.15 ± 3.0	0.73
100% fruit juice	1.2 ± 1.5 (0–5)	1.2 ± 1.4 (0–5)	−0.05 ± 1.4	0.82	1.2 ± 1.6 (0–7)	1.2 ± 1.4 (0–5)	−0.22 ± 1.9	0.94
**Total veggies ^b^**	5.4 ± 3.3 (1–17)	5.9 ± 2.7 (0–12)	0.45 ± 3.5	0.40	5.3 ± 4.4 (0–24)	5.8 ± 3.3 (1–17)	0.54 ± 5.4	0.50
White potatoes	1.6 ± 1.0 (0–4)	1.8 ± 1.5 (0–6)	0.17 ± 1.8	0.55	1.5 ± 1.4 (0–7)	1.7 ± 1.0 (0–5)	0.20 ± 1.6	0.40
Lettuce	2.0 ± 1.5 (0–7)	1.7 ± 1.1 (0–3)	−0.29 ± 1.3	0.17	1.6 ± 1.6 (0–7)	2.2 ± 1.5 (0–7)	0.54 ± 2.0	0.07
Other veggies	1.8 ± 1.6 (0–7)	2.3 ± 1.6 (0–7)	0.57 ± 2.0	0.07	2.1 ± 3.2 (0–21)	2.0 ± 1.5 (0–7)	−0.20 ± 3.7	0.72
**Total FV ^c^**	9.2 ± 4.9 (2–27)	9.9 ± 4.1 (3–20)	0.57 ± 4.5	0.41	8.1 ± 5.4 (1–28)	9.5 ± 4.6 (2–27)	1.50 ± 6.3	0.12
**VM score ^d^**	288.6 ± 45.7 (208–421)	289.8 ± 60.6 (92.5–423)	0.60 ± 46.9	0.47	276.9 ± 48.2 (179.5–388)	279.8 ± 46.5 (139–376)	3.00 ± 45.1	0.66
**HDBS ^e^**	6.5 ± 2.1 (1–12)	6.9 ± 1.6 (4–11)	0.42 ± 1.8	0.11	5.8 ± 2.1 (0–11)	6.6 ± 2.1 (1–12)	0.80 ± 2.1	0.01

Abbreviations: FV, fruits and vegetables; VM, Veggie Meter^®^; and HDBS, healthy dietary behavior score. Data are displayed as mean ± s.d. (range). Pre-test measures were taken two weeks prior to fathers starting the program. Post-test measures were taken within two weeks of fathers completing the six-week program. The reference period for dietary intakes and behaviors was the prior week. ^a^ Total fruit included fresh fruit and 100% fruit juice. ^b^ Total veggies included white potatoes, lettuce, and other veggies. ^c^ One total FV outlier was removed from the wait-listed control group pre-test data (*n* = 45). ^d^ Triplicate VM scans were collected at two separate times seven days apart and the averages were calculated. ^e^ Healthy dietary behavior scores were calculated by summing the responses of nine dietary behaviors. Mean change within-group for each outcome variable was evaluated using a paired samples *t*-test.

**Table 4:** Mixed analysis of variance summary table to evaluate differences in fathers’ weekly average Veggie Meter^®^ scores, total fruits and vegetables, and healthy dietary behavior scores between pre-test and post-test and between treatment and wait-listed control comparison groups.

Source of Variation	*df*	*F*-Value	*p*	*η* ^2^
**Veggie Meter^®^ scores**				
Group	1	1.22	0.27	0.01
Time	1	0.13	0.72	<0.01
Group × Time	1	0.06	0.81	<0.01
Total	175			
**Total FV ^a^**				
Group	1	0.85	0.36	<0.01
Time	1	3.01	0.08	0.01
Group × Time	1	0.58	0.45	<0.01
Total	174			
**HDBS**				
Group	1	1.32	0.25	0.01
Time	1	8.88	<0.01 *	0.02
Group × Time	1	0.70	0.41	<0.01
Total	175			

Abbreviations: *η*^2^, * *p* < 0.05. Abbreviations: *η*^2^, eta squared; *df*, degrees of freedom; FV, fruits and vegetables; and HDBS, healthy dietary behavior score. ^a^ One total FV outlier was removed from data analysis. Group differences accounted for very little in each of the outcomes.

**Table 5:** Mixed analysis of variance summary table for potential moderators of fathers’ weekly average healthy dietary behavior scores between pre-test and post-test and between treatment and wait-listed control comparison groups.

Source of Variation	*df*	*F*-Value	*p*	*η* ^2^
**Age**				
Group	1	0.79	0.37	<0.01
Time	1	7.96	<0.01 *	0.02
Group × Time × Age	1	0.10	0.75	<0.01
Total	175			
**Educational Attainment**				
Group	1	1.68	0.20	0.01
Time	1	8.40	<0.01 *	0.02
Group × Time × Education	1	3.41	0.07	<0.01
Total	175			
**Number of children in household**				
Group	1	0.91	0.34	<0.01
Time	1	7.85	<0.01 *	0.02
Group × Time × Children	1	1.93	0.17	<0.01
Total	175			

* *p* < 0.05. Abbreviations: *η*^2^, eta squared and *df*, degrees of freedom. Effect size estimates (*η*^2^) were very small indicating little differences in outcomes between groups.

## Data Availability

The data presented in this study are available on request from the corresponding author due to analyses are ongoing and findings will be reported in forthcoming manuscripts.

## Supplementary Materials

The following supporting information can be downloaded at: https://www.mdpi.com/article/10.3390/nu16081153/s1, File S1: HEPP Father Nutrition Survey, Table S1. Pre-test sociodemographic characteristics of 35 Mexican-heritage treatment participants and 46 wait-listed control comparisons participating in a six-week father-focused family-centered nutrition program, Table S2. Descriptive statistics of within-group mean change post-test for each program outcome for 35 treatment participants and 46 wait-listed control comparisons, and Table S3. Mixed analysis of variance summary table to evaluate differences in fathers’ weekly average Veggie Meter^®^ scores, total fruits and vegetables, and healthy dietary behavior scores between pre-test and post-test and between treatment (*n* = 35) and wait-listed control comparison (*n* = 46) groups. Table S4. Mixed analysis of variance summary table for potential moderators of fathers’ weekly average healthy dietary behavior scores between pre-test and post-test and between treatment (*n* = 35) and wait-listed control comparison (*n* = 46) groups.
