# Supplementary material for: An Evaluation of ¡Haz Espacio Para Papi!, a Culturally Tailored Nutrition and Physical Activity Pilot Program for Mexican-Heritage Fathers
Source: Nutrients. 2024 Apr 13;16(8):1153. doi: 10.3390/nu16081153 (PMC11053541; doi:10.3390/nu16081153)
Supplement: Supplementary file 1 [file nutrients-16-01153-s001.zip › File S1 HEPP Nutrition Survey.pdf]

Appendix A: HEPP Father Nutrition Survey

Start Time: _________ AM / PM **Date**: _____/_____/________ ***Promotora***: ________________________________

Participant ID #: _____-_______- _____-___________

Health for You and Your Family

Father Survey - Pre

F____________

<u>Diet</u> (see **answer grid A0**): I would like to ask you some questions about your food (meals and snacks). **Over the past week**, <u>how many times</u> did you eat the following items?

**1.**	**Snacks**	**How many times in the past week?**
	**a.** Chips	__________ times
	**b.** Popcorn	__________ times
	**c.** Nuts (almonds, peanuts, pecans, pistachios, etc.)	__________ times
	**d.** Ice cream (frozen yogurt, snow cone, popsicle, etc.)	__________ times
	**e.** Cookies	__________ times
	**f.** Cake	__________ times
	**g.** Pie	__________ times
	**h.** Pastries (sweet bread, donuts, cinnamon roll)	__________ times
	**i.** Candy (chocolates, lollipop, hard candy, etc.)	__________ times
**2.**	**Fruit**	**How many times in the past week?**
	**a.** Fresh fruit (any type)	__________ times
	**b.** 100% fruit juice	__________ times

COMMENTS:

Use A0 for questions 3-4

F____________

3.	Vegetables	How many times in the past week?
	**a.** White potatoes	__________ times
	**b.** White rice	__________ times
	**c.** Iceberg lettuce	__________ times
	**d.** Vegetables (not counting above items)	__________ times
**4.**	**Sugar-sweetened beverages**	**How many times in the past week?**
	**a.** Soda (Coke, Dr. Pepper, Sprite, Fanta, etc.)	__________ times
	**b.** Sugary drinks (*aguas frescas,* sweet tea, juice drinks, etc.)	__________ times
	**c.** Sport/energy drinks (Powerade, Gatorade, Red Bull, Monster, etc.)	__________ times

Nutrition knowledge (see **answer grids A1-A3, ✓ the answer**): I would like to ask you some questions about **nutrition in general**. Please choose the best answer (only one).

Question #5

What information can we learn from MyPlate?

☐ 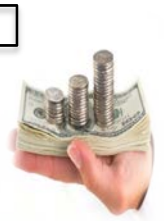 **How much food costs**	☐ 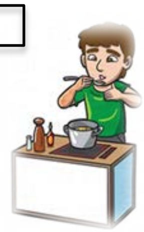 **What foods taste good**	☐ 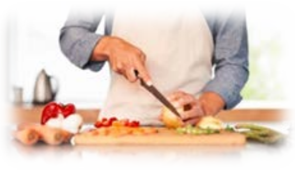 **How to prepare food**	☐ 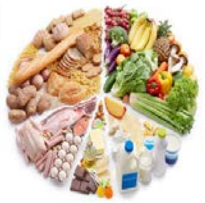 **How much to eat of different kinds of foods**	☐ 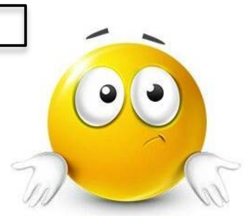 **I have never seen MyPlate**

COMMENTS:

Question #6

From which food group should you eat the most servings each day?

☐ 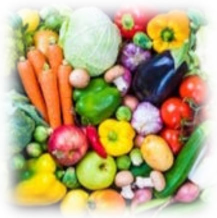	☐ 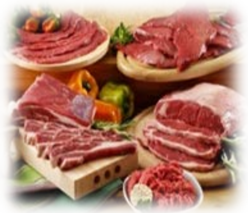	☐ 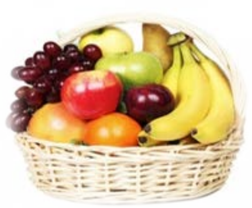	☐ 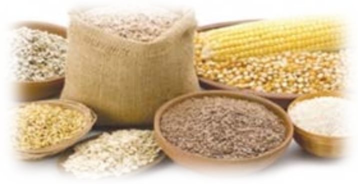
**Vegetables**	**Meat**	**Fruit**	**Grains**

Question #7

Why should we wash fruits and vegetables before we eat them?

☐ 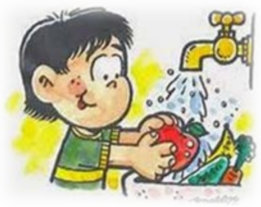	☐ 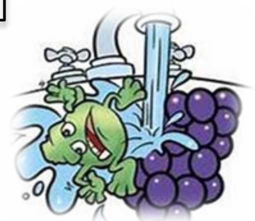	☐ 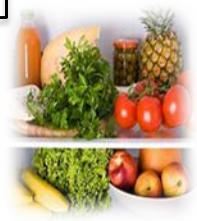	☐ 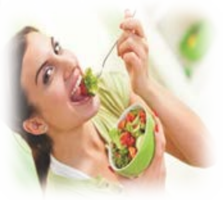
**To wash off the germs**	**Only if they are dirty**	**To keep them fresh**	**So they taste better**

Question #8

After cooking, how many maximum days should food be in the refrigerator?

☐ 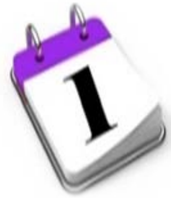	☐ 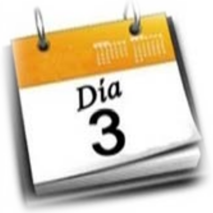	☐ 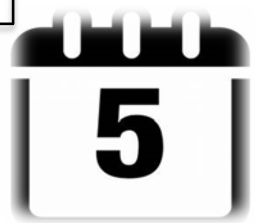	☐ 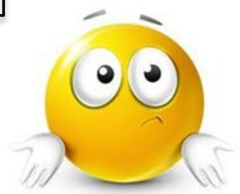
**1 day**	**3 days**	**5 days**	**Don't know**

COMMENTS:

Use A2 for questions 9-12

Question #9

*Why should we **not** eat foods that have been sitting out for more than 2 hours?*

☐ 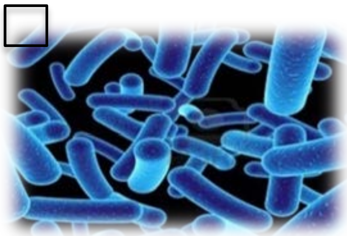 The vitamins break down and become bad for you	☐ 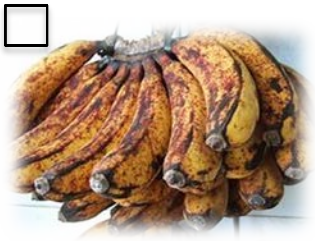 The color of the food changes	☐ 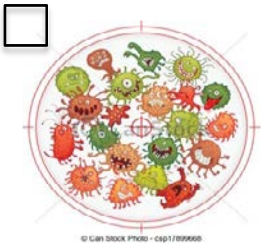 Germs grow on the food quickly	☐ 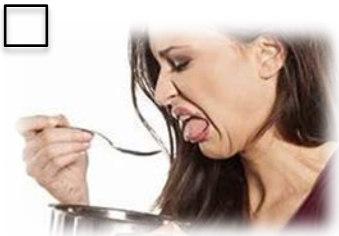 The food starts to taste bad

Question #10

With what should you fill half your plate?

☐ 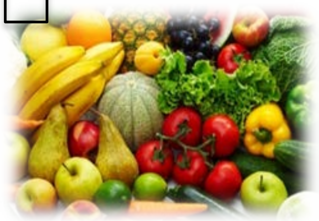 Fruits and vegetables	☐ 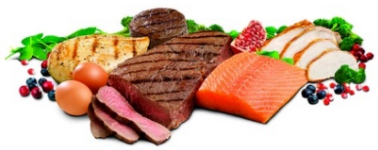 Protein	☐ 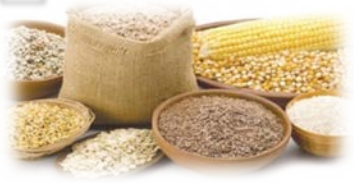 Grains	☐ 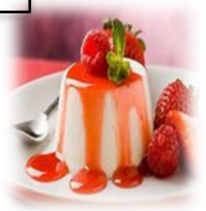 Dessert

Question #11

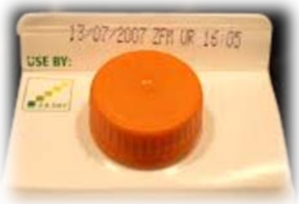

How often do you check the 'Use by' date on food packages?

☐ Never	☐ Sometimes	☐ Always

COMMENTS:

Question #12

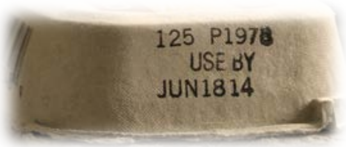

How often do you prepare/eat food past its ‘Use by’ date?

☐ Never	☐ Sometimes	☐ Always

Question #13

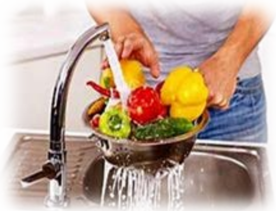

*How often do you wash fruits and vegetables that **<u>don’t need</u>** to be peeled before eating?*

☐ Never	☐ Sometimes	☐ Always

Question #14

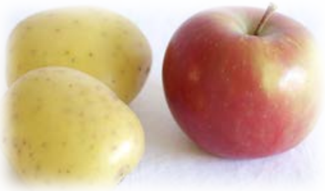

*How often do you wash fruits and vegetables that **<u>need</u>** to be peeled before eating?*

☐ Never	☐ Sometimes	☐ Always

COMMENTS:

Liking and Variety: For the following questions, I am going to ask you whether you have eaten any of the following fruits **in the past month** and **what you think about each of them** (see **answer grid A4, ✓ the answer and place X in box**).

Question #15

Fruits

**Fruit**<br><br>**What do you think about each of them?**	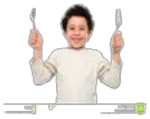<br>**Eaten in past month?**	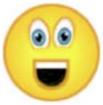<br>**Like**	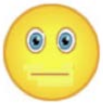<br>**Depends (Preparation)**	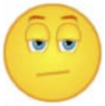<br>**Dislike**	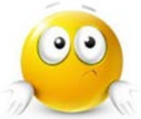<br>**Never tried**
**a. Peaches**<br>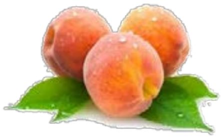	☐ Yes<br>☐ No	Like	Depends	Dislike	Never tried
**b. Strawberries**<br>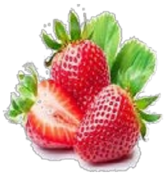	☐ Yes<br>☐ No	Like	Depends	Dislike	Never tried
**c. Guava**<br>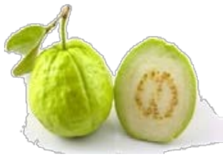	☐ Yes<br>☐ No	Like	Depends	Dislike	Never tried
**d. Lime/Lemon**<br>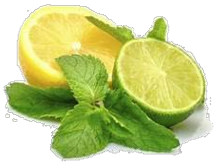	☐ Yes<br>☐ No	Like	Depends	Dislike	Never tried
**e. Tangerines**<br>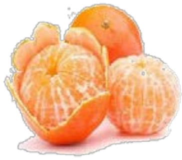	☐ Yes<br>☐ No	Like	Depends	Dislike	Never tried

COMMENTS:

**Fruit**<br><br>**What do you think about each of them?**	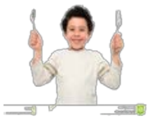<br>**Eaten in past month?**	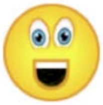<br>**Like**	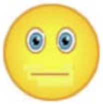<br>**Depends (Preparation)**	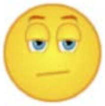<br>**Dislike**	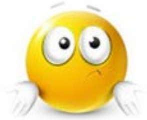<br>**Never tried**
**f. Mango**<br>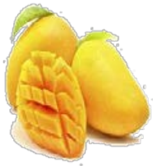	☐ Yes<br>☐ No	Like	Depends	Dislike	Never tried
**g. Apples**<br>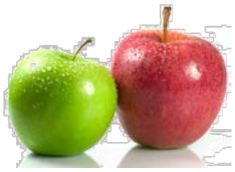	☐ Yes<br>☐ No	Like	Depends	Dislike	Never tried
**h. Melon**<br>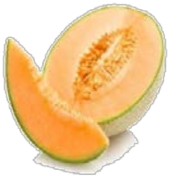	☐ Yes<br>☐ No	Like	Depends	Dislike	Never tried
**i. Oranges**<br>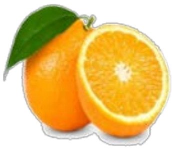	☐ Yes<br>☐ No	Like	Depends	Dislike	Never tried
**j. Other berries (blueberry, raspberry)**<br>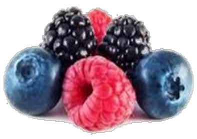	☐ Yes<br>☐ No	Like	Depends	Dislike	Never tried
**k. Papaya**<br>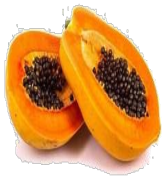	☐ Yes<br>☐ No	Like	Depends	Dislike	Never tried

COMMENTS:

**Fruit**<br><br>**What do you think about each of them?**	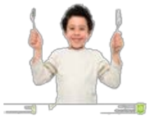<br>**Eaten in past month?**	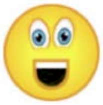<br>**Like**	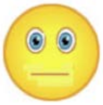<br>**Depends (Preparation)**	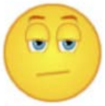<br>**Dislike**	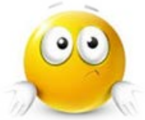<br>**Never tried**
**l. Pears**<br>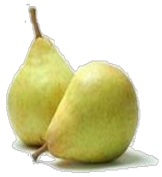	☐ Yes<br>☐ No	Like	Depends	Dislike	Never tried
**m. Pineapple**<br>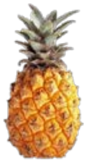	☐ Yes<br>☐ No	Like	Depends	Dislike	Never tried
**n. Banana**<br>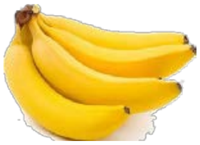	☐ Yes<br>☐ No	Like	Depends	Dislike	Never tried
**o. Plantain**<br>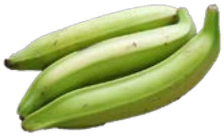	☐ Yes<br>☐ No	Like	Depends	Dislike	Never tried
**p. Grapefruit**<br>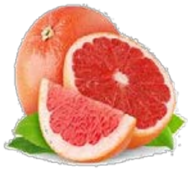	☐ Yes<br>☐ No	Like	Depends	Dislike	Never tried
**q. Grapes**<br>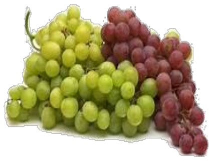	☐ Yes<br>☐ No	Like	Depends	Dislike	Never tried

COMMENTS:

**Fruit** What do you think about each of them?	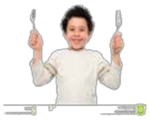 Eaten in past month?	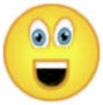 Like	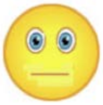 Depends (Preparation)	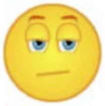 Dislike	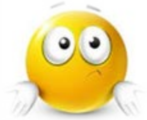 Never tried
r. 100% fruit juice - Apple 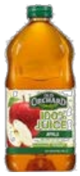	☐ Yes ☐ No	Like	Depends	Dislike	Never tried
s. 100% fruit juice - Orange 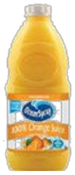	☐ Yes ☐ No	Like	Depends	Dislike	Never tried
t. 100% fruit juice - Other 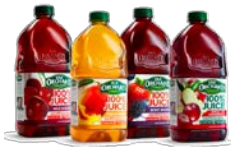	☐ Yes ☐ No	Like	Depends	Dislike	Never tried

COMMENTS:

Liking and Variety: For the following questions, I am going to ask you whether you have eaten any of the following vegetables **in the past month** and **what you think about each of them** (see **answer grid A4, ✔ the answer and place X in box**).

Question #16

Vegetables

**Vegetable**<br><br>**What do you think about each of them?**	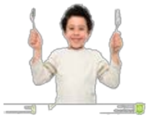<br>**Eaten in past month?**	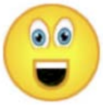<br>**Like**	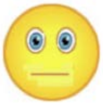<br>**Depends (Preparation)**	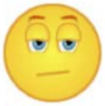<br>**Dislike**	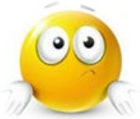<br>**Never tried**
**a. Avocado**<br>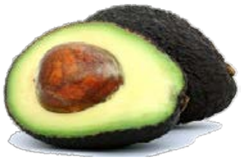	☐ Yes<br>☐ No	Like	Depends	Dislike	Never tried
**b. Garlic**<br>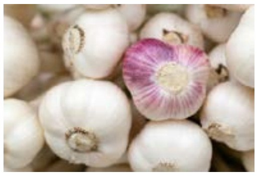	☐ Yes<br>☐ No	Like	Depends	Dislike	Never tried
**c. Celery**<br>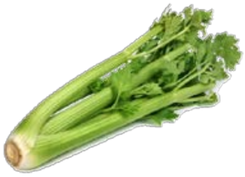	☐ Yes<br>☐ No	Like	Depends	Dislike	Never tried
**d. Beets**<br>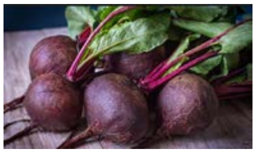	☐ Yes<br>☐ No	Like	Depends	Dislike	Never tried
**e. Broccoli**<br>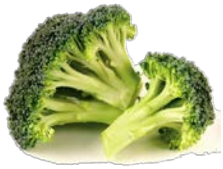	☐ Yes<br>☐ No	Like	Depends	Dislike	Never tried

COMMENTS:

**<u>Vegetable</u>**<br><br>**What do you think about each of them?**	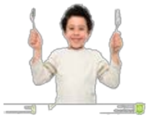<br>**Eaten in past month?**	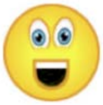<br>**Like**	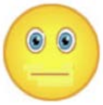<br>**Depends (Preparation)**	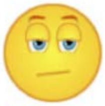<br>**Dislike**	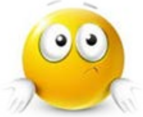<br>**Never tried**
**f. Squash (any kind)**<br>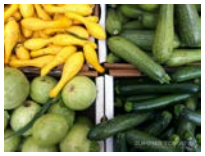	☐ Yes<br>☐ No	Like	Depends	Dislike	Never tried
**g. Pumpkin**<br>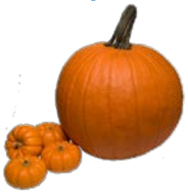	☐ Yes<br>☐ No	Like	Depends	Dislike	Never tried
**h. Sweet potato**<br>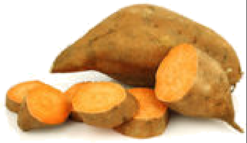	☐ Yes<br>☐ No	Like	Depends	Dislike	Never tried
**i. Onions (green, white, red)**<br>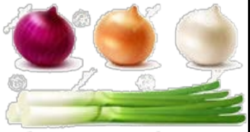	☐ Yes<br>☐ No	Like	Depends	Dislike	Never tried
**j. Peas (any kind)**<br>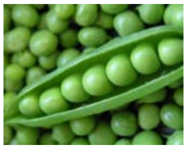	☐ Yes<br>☐ No	Like	Depends	Dislike	Never tried

COMMENTS:

**Vegetable**<br><br>**What do you think about each of them?**	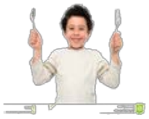<br>**Eaten in past month?**	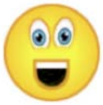<br>**Like**	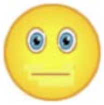<br>**Depends (Preparation)**	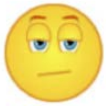<br>**Dislike**	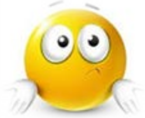<br>**Never tried**
**k. Bell pepper**<br>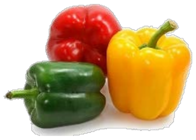	☐ Yes<br>☐ No	Like	Depends	Dislike	Never tried
**l. Hot peppers**<br>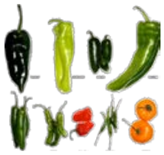	☐ Yes<br>☐ No	Like	Depends	Dislike	Never tried
**m. Cauliflower**<br>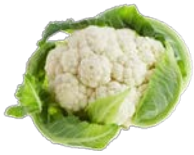	☐ Yes<br>☐ No	Like	Depends	Dislike	Never tried
**n. Green beans**<br>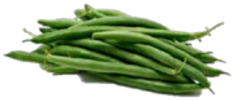	☐ Yes<br>☐ No	Like	Depends	Dislike	Never tried
**o. Corn**<br>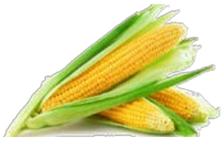	☐ Yes<br>☐ No	Like	Depends	Dislike	Never tried
**p. Spinach, chard or kale (dark green or leafy greens)**<br>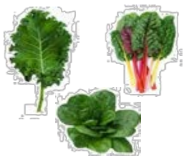	☐ Yes<br>☐ No	Like	Depends	Dislike	Never tried

COMMENTS:

**Vegetable**<br><br>**What do you think about each of them?**	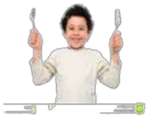<br>**Eaten in past month?**	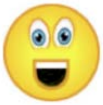<br>**Like**	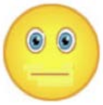<br>**Depends (Preparation)**	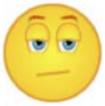<br>**Dislike**	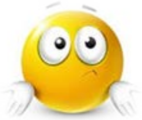<br>**Never tried**
**q. Beans (garbanzo, black beans, pinto)**<br>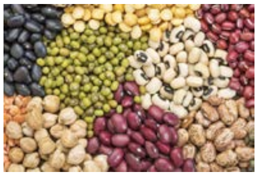	☐ Yes<br>☐ No	Like	Depends	Dislike	Never tried
**r. Jicama**<br>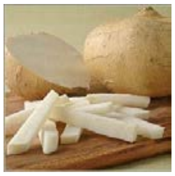	☐ Yes<br>☐ No	Like	Depends	Dislike	Never tried
**s. Iceberg lettuce**<br>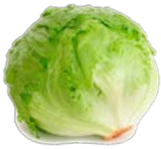	☐ Yes<br>☐ No	Like	Depends	Dislike	Never tried
**t. Romaine lettuce**<br>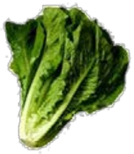	☐ Yes<br>☐ No	Like	Depends	Dislike	Never tried
**u. Nopales**<br>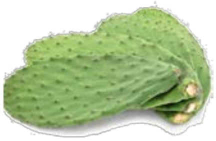	☐ Yes<br>☐ No	Like	Depends	Dislike	Never tried

COMMENTS:

<u>**Vegetable**</u><br><br>**What do you think about each of them?**	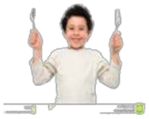<br>**Eaten in past month?**	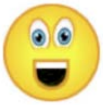<br>**Like**	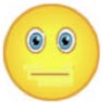<br>**Depends (Preparation)**	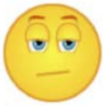<br>**Dislike**	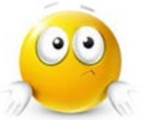<br>**Never tried**
**v. Potatoes – white**<br>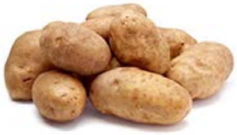	☐ Yes<br>☐ No	Like	Depends	Dislike	Never tried
**w. Cucumber**<br>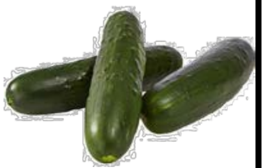	☐ Yes<br>☐ No	Like	Depends	Dislike	Never tried
**x. Cabbage**<br>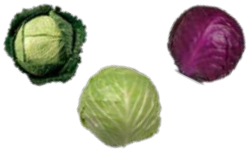	☐ Yes<br>☐ No	Like	Depends	Dislike	Never tried
**y. Tomato**<br>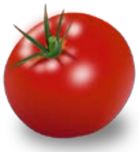	☐ Yes<br>☐ No	Like	Depends	Dislike	Never tried
**z. Yucca**<br>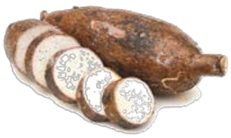	☐ Yes<br>☐ No	Like	Depends	Dislike	Never tried
**aa. Carrots**<br>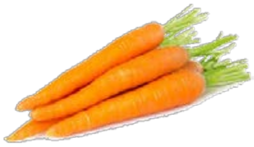	☐ Yes<br>☐ No	Like	Depends	Dislike	Never tried

COMMENTS:

Cooking Practices: I would like to ask you more about **cooking in general**. Please answer Yes, Sometimes, or No for each of the following questions [see **answer grid A5**] (**place X in box**).

	Yes	Sometimes	No
**17.** Do you feel limited by your lack of cooking knowledge?	Yes	Sometimes	No
**18.** Can you always manage to decide what you would like to eat at any given time?	Yes	Sometimes	No
**19.** When preparing/cooking food, are you confident that you can resolve unexpected results (didn't turn out as expected)?	Yes	Sometimes	No
**20.** When preparing/cooking food, is it easy for you to accomplish your desired results?	Yes	Sometimes	No
**21.** When preparing/cooking food, can you resolve most problems that come up?	Yes	Sometimes	No
**22.** Are you comfortable preparing food?	Yes	Sometimes	No
**23.** Do you know how to use the kitchen equipment you have?	Yes	Sometimes	No
**24.** Are you involved in daily meal preparation?	Yes	Sometimes	No
**25.** When you shop for food, do you know how you will use the ingredients you are purchasing?	Yes	Sometimes	No
**26.** Are you confident making meals from the ingredients you have on hand?	Yes	Sometimes	No
**27.** Before you start cooking, do you usually have a mental plan of all the steps you will follow?	Yes	Sometimes	No
**28.** When presented with two similar products to purchase, do you feel confident choosing between them?	Yes	Sometimes	No
**29.** When you go shopping, do you know where to find the ingredients you need to prepare a meal?	Yes	Sometimes	No

COMMENTS:

Cooking Thoughts: I would like to ask you about **your thoughts about cooking**. Please answer Yes, Sometimes, or No for each of the following questions [see **answer grid A5**] (**place X in box**).

	Yes	Sometimes	No
**30.** For you, is cooking an activity that makes you feel good?	Yes	Sometimes	No
**31.** For you, is cooking only something to get through as quickly as possible?	Yes	Sometimes	No
**32.** Do you feel that cooking brings you little enjoyment compared to other activities?	Yes	Sometimes	No
**33.** If you try making a new type of food and it does not come out right, do you usually try to make it again?	Yes	Sometimes	No
**34.** Do you think a lot about what you will cook or eat?	Yes	Sometimes	No
**35.** Do you prefer to spend your time on more important things than on cooking?	Yes	Sometimes	No
**36.** Normally, do you prefer to cook rather than to have someone else prepare the food?	Yes	Sometimes	No
**37.** Do you think food made at home is more affordable?	Yes	Sometimes	No
**38.** Do you get inspired to cook for other people, like your family or friends?	Yes	Sometimes	No
**39.** For you, is it a burden to have to cook for other people, like your family or friends?	Yes	Sometimes	No
**40.** Do you feel that you don't like to cook because it takes too much time?	Yes	Sometimes	No
**41.** Is cooking frustrating?	Yes	Sometimes	No
**42.** Do you find cooking tiring?	Yes	Sometimes	No
**43.** Does it take too much work to cook?	Yes	Sometimes	No

COMMENTS:

Cooking Time: I would like to ask you about your **time for cooking**. Please answer Yes, Sometimes, or No for each of the following questions [see **answer grid A5**] (**place X in box**).

	Yes	Sometimes	No
**44.** Would you like to have more time to plan meals?	Yes	Sometimes	No
**45.** Is it difficult for you to make time to prepare the food you would like to eat?	Yes	Sometimes	No
**46.** Do your family responsibilities prevent you from having the time to prepare meals?	Yes	Sometimes	No
**47.** Do your other activities (church, social events, volunteer time) prevent you from having the time to prepare meals?	Yes	Sometimes	No
**48.** Do your job responsibilities prevent you from having the time to prepare meals?	Yes	Sometimes	No

1st BREAK: ____________________________

Start Time: __________ AM / PM End Time: __________ AM / PM

Family Food Practices: I would like to ask you a few questions about **family meals**. The first five questions are about ________ and the rest are about you. **During the past month**, how often did you do the following [see **answer grid A6**] (**place X in box**)?

How often…?	Never	Once in a while	Almost every day
**49.** Did you talk to _________ about eating healthy foods?	Never	Once in a while	Almost every day
**50.** Did you take _________ with you to the store to buy groceries?	Never	Once in a while	Almost every day
**51.** Did you sit down to eat with _________?	Never	Once in a while	Almost every day
**52.** Did you ask _________ to help you prepare dinner?	Never	Once in a while	Almost every day
**53.** Did you ask _________ to help you plan meals?	Never	Once in a while	Almost every day

COMMENTS:

How often…?	Never	Once in a while	Almost every day
**54.** Did you prepare meals with everything homemade (basic ingredients)?	Never	Once in a while	Almost every day
**55.** Did you only use prepared foods (rotisserie chicken, pizza, bagged salad, prepared mashed potatoes, etc.) to make a meal?	Never	Once in a while	Almost every day
**56.** Did you combine both prepared foods (rotisserie chicken, pizza, bagged salad, prepared mashed potatoes, etc.) with homemade food items to make a meal?	Never	Once in a while	Almost every day
**57.** Did you reheat leftovers to use as all or part of another meal?	Never	Once in a while	Almost every day
**58.** Did you use leftovers from a home cooked meal to create a different dish?	Never	Once in a while	Almost every day
**59.** Did you use leftovers from a meal away from home (church, family, restaurant, etc.) to create a different dish?	Never	Once in a while	Almost every day
**60.** Did you eat breakfast away from home?	Never	Once in a while	Almost every day
**61.** Did you eat lunch away from home?	Never	Once in a while	Almost every day
**62.** Did you eat dinner away from home?	Never	Once in a while	Almost every day

COMMENTS:

Confidence: For the following questions, I am going to ask you how confident you feel in preparing the following food items to use them in your meals [see **answer grid A7**] (**place X in box**).

Question #63

Confidence

**Food Item**<br>**How confident do you feel in preparing/cooking the following food items to use them in your meals?**	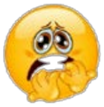<br>**Unconfident**	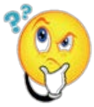<br>**Not sure**	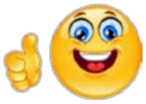<br>**Confident**
**a. Avocado**<br>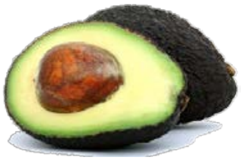	Unconfident	Not sure	Confident
**b. Celery**<br>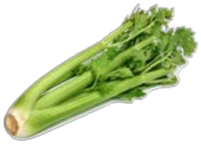	Unconfident	Not sure	Confident
**c. Beets**<br>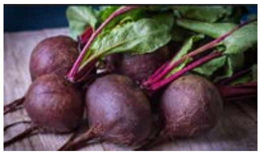	Unconfident	Not sure	Confident
**d. Broccoli**<br>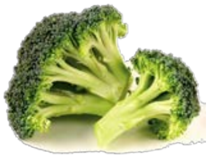	Unconfident	Not sure	Confident
**e. Pumpkin**<br>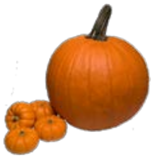	Unconfident	Not sure	Confident
**f. Squash (any kind)**<br>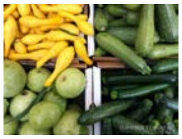	Unconfident	Not sure	Confident

COMMENTS:

Use A7 for these questions

**<u>Food Item</u>**<br>**How <u>confident</u> do you feel in preparing/cooking the following food items to use them in your meals?**	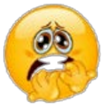<br>**Unconfident**	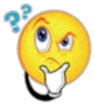<br>**Not sure**	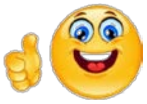<br>**Confident**
**g. Sweet potato**<br>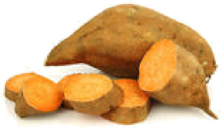	Unconfident	Not sure	Confident
**h. Onions (green, white, red)**<br>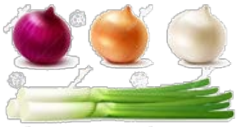	Unconfident	Not sure	Confident
**i. Peas (any kind)**<br>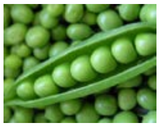	Unconfident	Not sure	Confident
**j. Cauliflower**<br>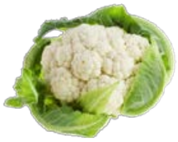	Unconfident	Not sure	Confident
**k. Green beans**<br>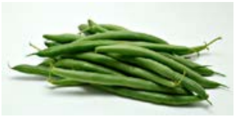	Unconfident	Not sure	Confident
**l. Corn**<br>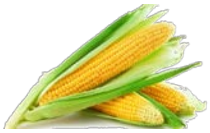	Unconfident	Not sure	Confident
**m. Spinach, chard or kale (dark green or leafy greens)**<br>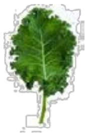 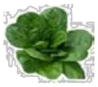 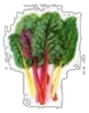	Unconfident	Not sure	Confident

COMMENTS:

**<u>Food Item</u>**<br>How <u>confident</u> do you feel in preparing/cooking the following food items to use them in your meals?	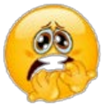<br>Unconfident	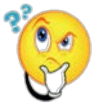<br>Not sure	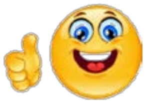<br>Confident
n. Beans(garbanzo, black beans, pinto)<br>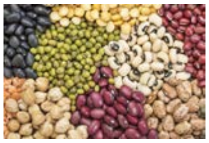	Unconfident	Not sure	Confident
o. Jicama<br>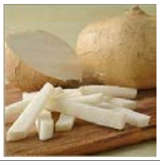	Unconfident	Not sure	Confident
p. Iceberg lettuce<br>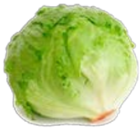	Unconfident	Not sure	Confident
q. Romaine lettuce<br>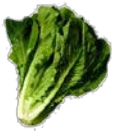	Unconfident	Not sure	Confident
r. Cucumber<br>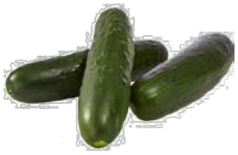	Unconfident	Not sure	Confident
s. Cabbage<br>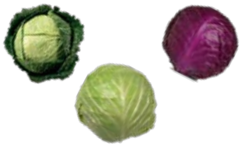	Unconfident	Not sure	Confident
t. Mixed vegetables (in stews, casseroles, or sides)<br>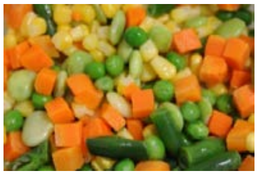	Unconfident	Not sure	Confident

COMMENTS:

Confidence: For the following questions, I am going to ask you how confident you feel in doing the following activities [see **answer grid A7**] (**place X in box**).

Question #64

Confidence

**How confident do you feel to…?**	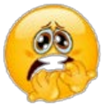 **Unconfident**	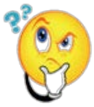 **Not sure**	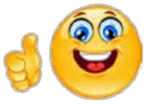 **Confident**
**a.** Wash your hands for at least 20 seconds before and during food preparation	Unconfident	Not sure	Confident
**b.** Plan or help decide which meals will be made at home	Unconfident	Not sure	Confident
**c.** Grocery shop for your family (e.g., choosing and purchasing foods at the store)	Unconfident	Not sure	Confident
**d.** Clean or wash fruits and vegetables before using and cooking	Unconfident	Not sure	Confident
**e.** Prepare fruits in different ways (juicing, pureeing, and chopping)	Unconfident	Not sure	Confident
**f.** Prepare a salad with homemade dressing	Unconfident	Not sure	Confident
**g.** Make beverages at home with vegetables and fruit **without added sugar**	Unconfident	Not sure	Confident
**h.** Prepare snacks at home for your children with vegetables and fruit	Unconfident	Not sure	Confident
**i.** Make vegetable-focused dishes at home	Unconfident	Not sure	Confident
**j.** Use herbs and spices (like basil, cilantro, garlic)	Unconfident	Not sure	Confident
**k.** Prepare meals with everything homemade (basic ingredients)	Unconfident	Not sure	Confident
**l.** Prepare foods with what you have at home	Unconfident	Not sure	Confident
**m.** Suggest a new dish or recipe to prepare at home	Unconfident	Not sure	Confident
**n.** Package up and store leftover food safely (for eating later)	Unconfident	Not sure	Confident
**o.** Eat vegetables as a snack	Unconfident	Not sure	Confident

COMMENTS:

Availability of Fruits and Vegetables: Thinking about **a normal week**, please answer Never, Once in a while, or Almost every day to each of the following questions [see **answer grid A8**] (**place X in box**).

Question #65

Availability

How often…?	Never	Once in a while	Almost every day
**a.** Do you eat meals that include vegetables as a family?	Never	Once in a while	Almost every day
**b.** Do you have fruit as dessert with meals?	Never	Once in a while	Almost every day
**c.** Do you encourage __________ to eat vegetables by eating them yourself?	Never	Once in a while	Almost every day
**d.** Is there fruit available in your home to have as a snack?	Never	Once in a while	Almost every day
**e.** Are there vegetables available in your home to have as a snack?	Never	Once in a while	Almost every day
**f.** Is there cut-up fruit at hand in the refrigerator to eat as a snack?	Never	Once in a while	Almost every day
**g.** Are there cut-up vegetables at hand in the refrigerator to eat as a snack?	Never	Once in a while	Almost every day
**h.** Is there fresh fruit on the counter, table, or somewhere else where you can easily get to it?	Never	Once in a while	Almost every day
**i.** Do you include __________ when you prepare a meal?	Never	Once in a while	Almost every day
**j.** Do you encourage __________ to eat more fruit?	Never	Once in a while	Almost every day
**k.** Do you encourage __________ to eat more vegetables?	Never	Once in a while	Almost every day
**l.** Do you help your wife/partner prepare a meal?	Never	Once in a while	Almost every day

COMMENTS:

How often…?	Never	Once in a while	Almost every day
**m.** Do you eat while watching TV or using electronics (computer, cell phone, tablet, etc.)?	Never	Once in a while	Almost every day
**n.** Does __________ eat while watching TV or using electronics (computer, cell phone, tablet, etc.)?	Never	Once in a while	Almost every day
**o.** Does your wife/partner eat while watching TV or using electronics (computer, cell phone, tablet, etc.)?	Never	Once in a while	Almost every day

For the next question, we're going to talk about **when you eat at home** [see **answer grid A9**] (**place X in box)**.

		Never	Once in a while	Almost every day	Where in your home?
**66.**	**a.** How often do you eat at home?	Never	Once in a while	Almost every day	
	**b.** How often do you eat somewhere else **in your home** (different from what you already mentioned)?	Never	Once in a while	Almost every day	

2nd BREAK: ______________________________

Start Time: __________ AM / PM End Time: __________ AM / PM

COMMENTS:

Child Activities: **During the past week**, has __________ done the following on his/her own? If so, how many times [see **answer grid A10**] (**place X in box**)?

Question #67

Child Activities

**Has ___________ done the following on his/her own?**	**No**	**Yes**	**If yes, how many times in the past week?**
**a.** Helped plan or decide which meals would be made at home	No	Yes	___________ times
**b.** Helped with grocery shopping	No	Yes	___________ times
**c.** Made snacks with fruit	No	Yes	___________ times
**d.** Made snacks with vegetables	No	Yes	___________ times
**e.** Helped prepare a meal	No	Yes	___________ times
**f.** Made a salad with fruits and vegetables	No	Yes	___________ times
**g.** Cut up fruit	No	Yes	___________ times
**h.** Cut up vegetables	No	Yes	___________ times
**i.** Measured ingredients	No	Yes	___________ times
**j.** Used a vegetable peeler	No	Yes	___________ times
**k.** Used a can opener	No	Yes	___________ times

COMMENTS:

**Has ___________ done the following on his/her own?**	**No**	**Yes**	**If yes, how many times in the past week?**
**l.** Read food labels	No	Yes	___________ times
**m.** Tried new foods	No	Yes	___________ times
**n.** Cleaned or washed fruits and vegetables before using	No	Yes	___________ times
**o.** Made beverages **without added sugar** with fruits and vegetables	No	Yes	___________ times
**p.** Helped with meal clean-up (e.g., clearing table, washing dishes)	No	Yes	___________ times

Your Activities: **During the past week**, have you done the following? If so, how many times [see **answer grid A10**] (**place X in box**)?

Question #68

Your Activities

**Have you done the following?**	**No**	**Yes**	**If yes, how many times in the past week?**
**a.** Washed your hands for at least 20 seconds before and during food preparation	No	Yes	___________ times
**b.** Planned or helped decide which meals will be made at home	No	Yes	___________ times
**c.** Grocery shopped for your family (e.g., choosing and purchasing foods at the store)	No	Yes	___________ times
**d.** Cleaned or washed fruits and vegetables before using and cooking	No	Yes	___________ times
**e.** Prepared fruits in different ways (juicing, pureeing, and chopping)	No	Yes	___________ times

COMMENTS:

Have you done the following?	No	Yes	If yes, how many times in the past week?
**f.** Prepared a salad with homemade dressing	No	Yes	___________ times
**g.** Made beverages at home with vegetables and fruit **without added sugar**	No	Yes	___________ times
**h.** Prepared snacks at home for your children with vegetables and fruit	No	Yes	___________ times
**i.** Made vegetable-focused dishes at home	No	Yes	___________ times
**j.** Used herbs and spices (like basil, cilantro, garlic)	No	Yes	___________ times
**k.** Prepared meals with everything homemade (basic ingredients)	No	Yes	___________ times
**l.** Prepared foods with what you have at home	No	Yes	___________ times
**m.** Suggested a new dish or recipe to prepare at home	No	Yes	___________ times
**n.** Packaged up and stored leftover food safely (for eating later)	No	Yes	___________ times
**o.** Took lead on preparing a meal for your family	No	Yes	___________ times
**p.** Helped with meal clean-up (e.g., clearing table, washing dishes)	No	Yes	___________ times
**q.** Ate vegetables as a snack	No	Yes	___________ times

COMMENTS:

<u>Your Cooking Confidence</u>: I would like to ask you about <u>how confident</u> you feel to cook [see **answer grid A11**] (**place X in box**).

Question #69

Your Cooking Confidence

You	No	Yes
**a.** Do you feel confident preparing a meal for your family?	No	Yes
**b.** Are you very happy to cook for yourself?	No	Yes
**c.** Do you feel confident that what you cook will turn out as planned?	No	Yes
**d.** Are you willing to eat healthy?	No	Yes
**e.** Are you willing to cook?	No	Yes
**f.** Do you have good cooking skills?	No	Yes
**g.** Do you like to try new foods?	No	Yes

<u>Family Influence</u>: I would like to ask about your and your family's **use of vegetables**.

**70.** What vegetables do <u>you</u> like?	
**71.** What vegetables do <u>you</u> **not** like?	

COMMENTS:

**72.** What vegetables does your wife/partner like?	
**73.** What vegetables does your wife/partner **not** like?	
**74.** What vegetables does _________ like?	
**75.** What vegetables does _________ **not** like?	
**76.** What would be your wife/partner's reaction to having a vegetable prepared in a different way?	
**77.** What would be _________'s reaction to having a vegetable prepared in a different way?	
**78.** Who would make it easy for _________ to eat vegetables?	
**79.** Who would make it difficult for _________ to eat vegetables?	

COMMENTS:

**80.** What are the **benefits** of having vegetables in your home?	
**81.** What would help you to have vegetables in your home?	

Food Security: Now I am going to ask you about the foods that you have in your home (✓ **the answer**).

**82.** In the **past month**, did you ever worry that you would run out of food before you got money to buy more?	☐ Yes	☐ No	☐ Refused to answer
**83.** In the **past month**, did your household ever run out of food?	☐ Yes	☐ No	☐ Refused to answer
**84.** In the **past month**, did your household ever run out of the foods needed to make a complete meal?	☐ Yes	☐ No	☐ Refused to answer
**85.** In the **past month**, did you ever use a low-cost food (such as macaroni and cheese, ramen noodles, peanut butter, or little or no meat, vegetables, or fruit) because there wasn't enough food for everyone?	☐ Yes	☐ No	☐ Refused to answer
**86.** In the **past month**, did you or another adult in your household ever eat less or skipped meals because there wasn't enough food for everyone?	☐ Yes	☐ No	☐ Refused to answer
**87.** In the **past month**, did you ever eat less than you wanted to because there wasn't enough food for everyone?	☐ Yes	☐ No	☐ Refused to answer
**88.** In the **past month**, were you ever hungry but didn't eat because there wasn't enough food for everyone?	☐ Yes	☐ No	☐ Refused to answer
**89.** In the **past month**, did you or another adult in your household ever **not eat** for a whole day because there wasn't enough food for everyone?	☐ Yes	☐ No	☐ Refused to answer
**90.** In the **past month**, did you or another adult in your household ever have to **eat fewer meals** for a whole day because there wasn't enough food for everyone?	☐ Yes	☐ No	☐ Refused to answer

COMMENTS:

**91.** In the **past month**, did you ever have to choose between paying bills and buying food?	☐ Yes	☐ No	☐ Refused to answer
**92.** In the **past month**, did you ever eat less food so that someone else in the family could eat more?	☐ Yes	☐ No	☐ Refused to answer
**93.** In the **past month**, did you or other adults in your household ever borrow money to buy food?	☐ Yes	☐ No	☐Refused to answer

BACKGROUND

	**Question**	**Answer**	
**94.**	What is your date of birth?	______/_____/______<br>month day year	
**95.**	What was the highest grade you completed in school?	☐ College degree<br>☐ Some college<br>☐ High school degree	☐ GED<br>☐ Some school<br>☐ Didn't go to school
**96.**	At this moment, what is your marital status?	☐ Married<br>☐ Living with partner – not married	
**97.**	At this moment, what are the ages of people living in your home (**not including yourself**)?	**Person**	**Ages**
		Adults<br>(**18 or older**)	
		Children<br>(**17 or younger**)	

End Time: ________________ AM / PM

COMMENTS:
