# Supplementary material for: An Evaluation of ¡Haz Espacio Para Papi!, a Culturally Tailored Nutrition and Physical Activity Pilot Program for Mexican-Heritage Fathers
Source: Nutrients. 2024 Apr 13;16(8):1153. doi: 10.3390/nu16081153 (PMC11053541; doi:10.3390/nu16081153)
Supplement: Supplementary file 1 [file nutrients-16-01153-s001.zip › Table S1.pdf]

Table S1. Pre-test sociodemographic characteristics of 35 Mexican-heritage treatment participants and 46 wait-listed control comparisons participating in a six-week father-focused family-centered nutrition program.

**Sociodemographic characteristic**	**Treatment (n = 35)**	**Control comparison (n = 46)**
	Mean ± s.d. (range) or n (%)	
Age	38.9 ± 7.1 (28–59)	38.7 ± 8.9 (26–58)
Body mass index[a]	30.1 ± 5.4 (22.1–46.1)	31.3 ± 5.5 (22.1–49.7)
Marital status		
Married	23 (65.7)	25 (54.4)
Living with spouse – not married	12 (34.3)	21 (45.7)
Education attainment		
Didn't go to school	1 (2.9)	1 (2.3)
Some school	24 (68.6)	34 (73.9)
GED or HS diploma	8 (22.8)	9 (19.6)
Some college	2 (5.7)	1 (2.2)
College degree	0 (0.0)	1 (2.2)
Total residents in household	7.1 ± 2.1 (4–13)	7.1 ± 2.0 (4–13)
Number of adults[b]	2.6 ± 1.0 (2–5)	2.7 ± 1.0 (2–6)
Number of children[c]	4.5 ± 1.9 (2–8)	4.4 ± 1.9 (2–9)
Ages of adults in household (years of age)	36.4 ± 11.4 (18–79)	37.3 ± 12.3 (26–77)
Ages of children in household (years of age)	9.0 ± 4.2 (3 mo–17)	9.3 ± 4.2 (3 mo–17)

Abbreviations: Abbreviations: GED, general education development; HS, high school. *Promotoras* gathered pre-test (baseline) sociodemographic characteristics from program participants and wait-list control comparisons two weeks prior to participants starting the program. [a]Body mass index was calculated using kg/m^2. [b]Adults were defined as ≥18 years of age. [c]Children were defined as ≤17 years of age.
