# Supplementary material for: An Evaluation of ¡Haz Espacio Para Papi!, a Culturally Tailored Nutrition and Physical Activity Pilot Program for Mexican-Heritage Fathers
Source: Nutrients. 2024 Apr 13;16(8):1153. doi: 10.3390/nu16081153 (PMC11053541; doi:10.3390/nu16081153)
Supplement: Supplementary file 1 [file nutrients-16-01153-s001.zip › Table S2.pdf]

Table S2. Descriptive statistics of within-group mean change at post-test for each program outcome for 35 treatment participants and 46 wait-listed control comparisons.

Measure	Treatment (n = 35)				Control comparison (n = 46[e])			
	Pre-test	Post-test	Mean change	*P* value	Pre-test	Post-test	Mean change	*P* value
**Total fruit[a]**	3.7 ± 2.4 (0–10)	4.0 ± 2.4 (1–10)	0.23 ± 1.8	0.47	3.5 ± 3.2 (0–16)	3.7 ± 2.4 (0–10)	0.13 ± 3.6	0.81
Fresh fruit	2.5 ± 1.6 (0–7)	2.8 ± 1.5 (0–8)	0.31 ± 1.6	0.25	2.3 ± 2.6 (0–16)	2.5 ± 1.6 (0–7)	0.15 ± 3.0	0.73
100% fruit juice	1.2 ± 1.5 (0–5)	1.2 ± 1.4 (0–5)	-0.09 ± 1.3	0.70	1.2 ± 1.6 (0–7)	1.2 ± 1.4 (0–5)	-0.22 ± 1.9	0.94
**Total veggies[b]**	5.2 ± 3.3 (1–17)	5.7 ± 2.7 (0–12)	0.51 ± 3.7	0.42	5.3 ± 4.4 (0–24)	5.8 ± 3.3 (1–17)	0.54 ± 5.4	0.50
White potatoes	1.5 ± 1.0 (0–4)	1.8 ± 1.5 (0–6)	0.29 ± 1.9	0.39	1.5 ± 1.4 (0–7)	1.7 ± 1.0 (0–5)	0.20 ± 1.6	0.40
Lettuce	2.0 ± 1.5 (0–7)	1.7 ± 1.1 (0–3)	-0.29 ± 1.4	0.23	1.6 ± 1.6 (0–7)	2.2 ± 1.5 (0–7)	0.54 ± 2.0	0.07
Other veggies	1.7 ± 1.6 (0–7)	2.2 ± 1.7 (0–7)	0.51 ± 2.1	0.15	2.1 ± 3.2 (0–21)	2.0 ± 1.5 (0–7)	-0.20 ± 3.7	0.72
**Total FV**	9.0 ± 5.0 (2–27)	9.7 ± 4.2 (3–20)	0.74 ± 4.7	0.35	8.1 ± 5.4 (1–28)	9.5 ± 4.6 (2–27)	1.50 ± 6.3	0.12
**VM score[c]**	289.3 ± 46.7 (208–421)	292.6 ± 56.0 (183–423)	3.26 ± 36.8	0.60	276.9 ± 48.2 (179.5–388)	279.8 ± 46.5 (139–376)	3.00 ± 45.1	0.66
**HDBS[d]**	6.4 ± 2.1 (1–12)	6.7 ± 1.6 (4–11)	0.34 ± 1.9	0.30	5.8 ± 2.1 (0–11)	6.6 ± 2.1 (1–12)	0.80 ± 2.1	0.01

Abbreviations: FV, fruits and vegetables; VM, Veggie Meter®; HDBS, healthy dietary behavior score. Data are displayed as mean ± s.d. (range). Pre-test measures were taken two weeks prior to fathers starting the program. Post-test measures were taken within two weeks of fathers completing the six-week program. Reference period for dietary intakes and behaviors was the prior week. [a]Total fruit included fresh fruit and 100% fruit juice. [b]Total veggies included white potatoes, lettuce, and other veggies. [c]Triplicate VM scans were collected at two separate times seven days apart and averages were calculated. [d]Healthy dietary behavior scores were calculated by summing responses of nine dietary behaviors. Mean change within group for each outcome variable was evaluated using a paired samples t-test (mean change ± s.d.). [e]One total FV outlier was removed from the wait-listed control group pre-test data (n = 45).
