# Supplementary material for: An Evaluation of ¡Haz Espacio Para Papi!, a Culturally Tailored Nutrition and Physical Activity Pilot Program for Mexican-Heritage Fathers
Source: Nutrients. 2024 Apr 13;16(8):1153. doi: 10.3390/nu16081153 (PMC11053541; doi:10.3390/nu16081153)
Supplement: Supplementary file 1 [file nutrients-16-01153-s001.zip › Table S3.pdf]

Table S3. Mixed analysis of variance summary table to evaluate differences in fathers' weekly average Veggie Meter® scores, total fruits and vegetables, and healthy dietary behavior scores between pre-test and post-test and between treatment (n – 35) and wait-listed control comparison (n = 46) groups.

Source of variation	*df*	*F*-value	*P*	η^{2b}
**Veggie Meter® scores**				
Group	1	1.59	0.21	0.02
Time	1	0.45	0.51	<0.01
Group*Time	1	0.00	1.0	<0.01
Total	161			
**Total FV[a]**				
Group	1	0.38	0.54	<0.01
Time	1	3.03	0.09	<0.01
Group*Time	1	0.33	0.33	<0.01
Total	160			
**HDBS**				
Group	1	0.67	0.42	<0.01
Time	1	6.36	**0.01***	0.02
Group*Time	1	1.03	0.31	<0.01
Total	161			

Abbreviations: η^2, eta squared; *df*, degrees of freedom; FV, fruits and vegetables; HDBS, healthy dietary behavior score. [a]One total FV outlier was removed from data analysis. [b]Group differences accounted for very little in each of the outcomes.
