# Supplementary material for: An Evaluation of ¡Haz Espacio Para Papi!, a Culturally Tailored Nutrition and Physical Activity Pilot Program for Mexican-Heritage Fathers
Source: Nutrients. 2024 Apr 13;16(8):1153. doi: 10.3390/nu16081153 (PMC11053541; doi:10.3390/nu16081153)
Supplement: Supplementary file 1 [file nutrients-16-01153-s001.zip › Table S4.pdf]

Table S4. Mixed analysis of variance summary table for potential moderators of fathers' weekly average healthy dietary behavior scores between pre-test and post-test and between treatment (n = 35) and wait-listed control comparison (n = 46) groups.

Source of variation	*df*	*F*-value	*P*	η^{2a}
**Age**				
Group	1	0.28	0.59	<0.01
Time	1	5.63	**0.02***	0.02
Group*Time*Age	1	0.17	0.68	<0.01
Total	161			
**Educational Attainment**				
Group	1	1.18	0.28	0.01
Time	1	7.06	**<0.01***	0.02
Group*Time*Education	1	2.73	0.10	<0.01
Total	161			
**Number of children in household**				
Group	1	0.37	0.54	<0.01
Time	1	5.37	**0.02***	0.02
Group*Time*Children	1	0.87	0.35	<0.01
Total	161			

Abbreviations: η^2, eta squared; *df*, degrees of freedom. [a]Effect size estimates (η^2) were very small indicating little differences in outcomes between groups. * p<0.05.
